# Investigation of Waste Electrical Power Plant Oil as a Rejuvenating Agent for Reclaimed Asphalt Binders and Mixtures

**DOI:** 10.3390/ma15144811

**Published:** 2022-07-10

**Authors:** Eman M. El-labbad, Usama Heneash, Sherif M. El-Badawy

**Affiliations:** 1 Civil Engineering Department, Faculty of Engineering, KafrEl Sheikh University, Kafr El Sheikh 33516, Egypt; emanellabad@yahoo.com (E.M.E.-l.); usama.heneash@eng.kfs.edu.eg (U.H.); 2 Highway and Airport Engineering Laboratory, Public Works Engineering Department, Faculty of Engineering, Mansoura University, 60 Elgomhoria St., Mansoura 35516, Egypt

**Keywords:** RAP, WEO, master curve, FTIR, dynamic modulus, flow number

## Abstract

One of the main difficulties with employing recycled asphalt pavement (RAP) in hot mix asphalt (HMA) is bitumen aging; hence, the percentage of RAP in the HMA is limited. This research evaluates the rheological properties of the RAP binder and the performance of HMA containing high RAP content using waste engine oil (WEO) from an Electrical Power Plant as a rejuvenator. The rheological and microstructural properties of the RAP binder and rejuvenated RAP binder were determined in the laboratory. Both the recycled and rejuvenated recycled mixes were tested for Marshall stability, indirect tensile strength, dynamic modulus (E*), and flow number tests. The RAP binder was recovered using two different processes: rotavapor distillation followed by centrifugation (RCRD) and column distillation without centrifugation (RNCCD). The optimal WEO percentages for the RCRD and RNCCD recovery procedures were 0.5% and 3%, respectively. The Marshall test results revealed that adding WEO to the recycled mix enhanced its stability and flow compared to the control mix. The rejuvenated mix containing recovered binder from the RCRD recovery process was found to be better than the rejuvenated mix containing recovered binder from the RNCCD recovery process. The rejuvenated recycled mixes outperformed the recycled mix in terms of moisture resistance, which was evidenced by tensile strength ratio values of 0.88, 0.90, and 0.91 for the control and 0.5% and 3% WEO modified mixes, respectively. Finally, the results of dynamic modulus and flow number testing revealed that the rejuvenated mixes had a modest drop in both the dynamic modulus and flow number compared to the non-rejuvenated mix.

## 1. Introduction

Bitumen is one of the most often utilized bonding materials for asphalt pavement [1]. Approximately 95% of globally produced bitumen each year is used by the paving industry sector [2]. Declining supplies of locally accessible good-quality aggregates, rising waste disposal concerns, and rising bitumen prices have resulted in higher usage of recycled asphalt pavement (RAP) in the construction of infrastructure projects [3]. Recently, pavement recycling has gained prominence in developing countries. The recycling of pavements includes the incorporation of discarded or damaged materials for new pavement construction [4]. The mix design process takes into account the amount of bitumen in RAP when the amount of RAP used in hot mix asphalt (HMA) reaches 25% or more [5]. Moisture damage, fatigue, and thermal cracking are almost certain to occur in recycled high-RAP content mixes, where they become stiff due to the aged bitumen. Consequently, characterization of the RAP binder and aggregate is a crucial step in the design [6,7].

Earlier studies have demonstrated that after the asphalt pavement life cycle has ended, the bitumen and aggregate from the old pavement are still significant resources [8]. Aging of the RAP binder is the major concern that has limited the use of RAP content to just low percentages [9]. Bitumen’s aging problem contributes to premature pavement cracking. Consequently, the cost of restoring and maintaining bituminous pavements rises. The term “asphalt ageing” most likely relates to changes in the rheological characteristics of the RAP binder as a result of chemical and temperature changes [10,11]. The aging of the RAP binder considerably affects its rheological properties, thereby affecting the overall asphalt pavement performance [12]. A common approach to recovering the properties of the RAP binder to a state resembling virgin bitumen is through rejuvenation [13]. Chemical additives, rejuvenating (recycling) agents, and softening agents are widely available and can be added to pavements containing RAP. Recycling agents are an appropriate way of lowering the stiffness and increasing the percent of RAP in the mix. The performance of recycling agents is primarily influenced by their original sources [14]. The main distinction between softening and rejuvenating compounds is that a rejuvenating agent can retain the chemical structure of aged bitumen, whilst a softening agent reduces the aged binder’s overall viscosity [15,16]. Rejuvenators are biological additives with high maltene content, which appear to offset the higher concentration of insoluble asphaltenes in the aged binder [17], thus decreasing the stiffness and increasing the phase angle of the resulting rejuvenated binder [18]. Recently, the use of rejuvenators has become popular, proving to be a feasible alternative with higher-RAP materials. Several researchers have looked at the effects of different rejuvenators on the rheological properties of bitumen. It has been demonstrated that rejuvenators significantly reduce stiffness, leading to improved resistance to fatigue cracking [19,20]. Recently, producing asphalt rejuvenators from waste oils has gained wide acceptance. The processes of filtering, sorting, and blending are frequently used in the production of rejuvenators [21]. The rejuvenation mechanism is accomplished by reinstating the colloid structure of bitumen and improving the movement of molecules [22]. Bio-oils can rejuvenate the aged binder. According to the latest research findings, they can be utilized to soften hard aged bitumen and lessen the stiffness of binders and high-RAP content mixes [23,24]. Furthermore, a recent analysis observed that the addition of bio-binder enhanced the asphalt binder grade at high temperatures while lowering it at low temperatures [25]. For example, due to its lower cost and reduced environmental problems, bio-waste cooking oil is useful. However, it has some limitations that might restrict its pertinence, such as low-temperature properties and a tendency for oxidation [26]. The use of WEO has attracted significant interest in response to trash management, environmental issues, and financial benefits [27]. When WEO and RAP bitumen are combined together, coherent bonding is achieved through the alteration of the components and the rejuvenation of the aged binder [28,29,30]. WEO usually includes contaminants such as moisture, dust, diluents, grime, and metal fragments from engine wear due to the combustion process [31]. The addition of WEO to the extracted RAP binder displays effectiveness in softening and allows for the calculation of the dose needed to meet the binder specifications [32]. Table 1 summarizes the most relevant literature studies on the use of waste engine oil as a rejuvenator of binders and asphalt mixes. The main outcome of these literature studies is that the characteristics of aged binders/mixes, including RAP, can be improved using different types of rejuvenators from waste. One of the probable urban waste sources that can be used to revitalize the RAP binder is waste engine oil (WEO) from Power Plant Engines. Waste Electrical Power Plant oil is a kind of WEO. However, because the nature of the Electrical Power Plant means that it must run continuously, Electrical Power Plant oils usually have certain properties to keep engines running safely. Thus, it is a heavy-duty chemically altered oil blend that maintains the proper oil viscosity under extreme operating conditions compared to automobile engine oils [33,34]. In this study, a semi-synthetic waste oil that was originally a mineral oil blended with synthetic oil to improve engine performance and reduce engine friction was used in the analysis. Usually, after use (recycled), this oil will be contaminated with a low percentage of impurities due to engine wear during operation. A previous study reported a considerable amount of aluminum in waste engine oils collected from automobile shops and attributed this to engine wear [32]. Furthermore, the optimal amount of the rejuvenator depends mainly on the properties of the aged bitumen (AB) and virgin bitumen and the type of rejuvenator. Moreover, very limited studies have been conducted on local mixtures containing high RAP content and waste rejuvenators in Egypt.

Thus, the aim of this research was to evaluate the effect of using locally available WEO from Power Plant Engines as a rejuvenator on the chemical and rheological properties of the RAP binder from an old pavement in Egypt using two extraction methods for the RAP binder. It also investigated how the WEO affects the performance of typical HMA with high RAP content in terms of the dynamic modulus, flow number, and moisture susceptibility. This research article is divided into five different sections. This section covers a comprehensive literature review of relevant studies on the use of WEO as a rejuvenator for aged binders and ends with the research motivation and objectives. The second section describes the investigated materials, which are the waste electrical power plant oil, RAP binder, and aggregate. The third section presents the details of the conducted testing program on both the binder and mixture in order to achieve the research objectives. The fourth section is the discussion and analysis of the test results, and then the research conclusions are outlined in the fifth and last section of the article.

## 2. Materials

Virgin bitumen, virgin aggregate, RAP, and WEO were used in the study. The Suez Oil company supplied 60/70 penetration grade bitumen for this research with a softening point of 45 °C. This is the typical binder grade used in Egypt. Table 2 displays the properties of the virgin binder. The virgin aggregate was crushed dolomite stone. The specific gravity of fine and coarse aggregates is 2.45 and 2.68, respectively, and the specific gravity of the mineral filler is 2.68. Moreover, Los Angeles Abrasion and the water absorption values for coarse aggregates are 37.3 and 3.4, respectively. The gradation of the used aggregate is shown in Table 3. All properties meet the specifications of the Egyptian Code of Practice (ECP) [43]. A cold milling machine was used to collect RAP from a major road in Egypt’s Al-Daqahliyah province, which has been in service for 14 years. The RAP gradation is also shown in Table 3. The WEO was sourced from Kafr El-Sheikh Electrical Power Plant, Egypt, with a specific gravity of 0.87 and a flash point of 195 °C. The bitumen content of the RAP was measured by using centrifuge extraction, and the bitumen content was found to be 5% by the weight of the mixture.

## 3. Experimental Work

**Figure 1:**
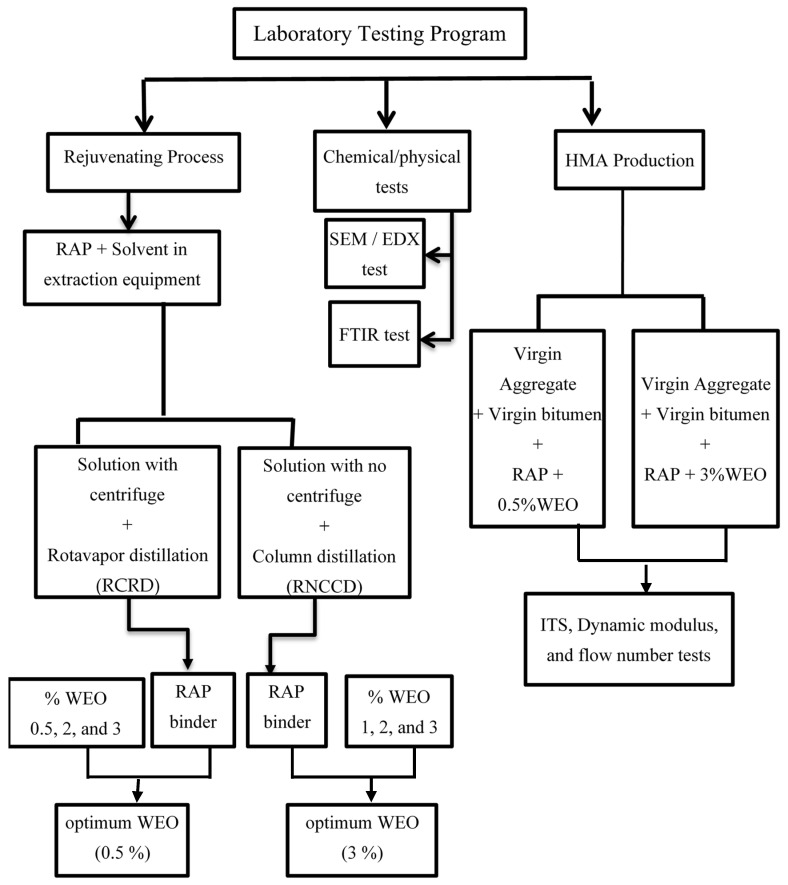
Experimental program flow chart.

The experimental work was separated into four main tasks in order to achieve the objectives of this research. The first one was bitumen extraction from RAP, followed by a recovery process, which was based mostly on concentrating the bitumen solution (bitumen and solvent) by distillation. The second task included the characterization of the RAP binder and the virgin binder through traditional bitumen tests and determining the optimal percentage of the selected rejuvenator. The third task was the design of a conventional asphalt mix with high RAP content using the Marshall mix design method, and the last task involved laboratory testing to assess the rejuvenated mixtures’ performance in comparison with the control mix. It is worth noting that Egypt and many countries in the Middle East still use the conventional Marshall mix design method along with the penetration grading system for binder characterization [44]. Figure 1 outlines the experimental testing program.

As shown in Figure 1, the extraction process included adding the methylene chloride to RAP materials and then collecting the solution. After the extraction process, it was observed that the solution contained a substantial amount of impurities, which influenced the bitumen test results. As a result, a centrifugal process was carried out to remove impurities.

**Figure 2:**
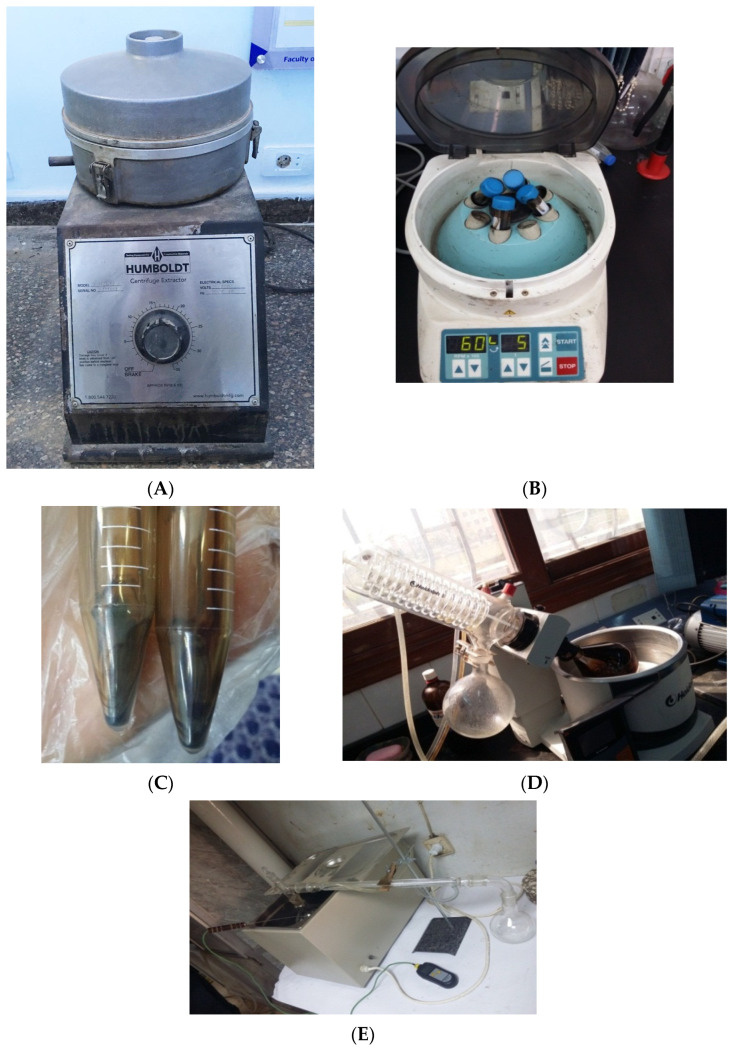
Extraction, distillation, recovery, and centrifugation. (**A**) Extraction device, (**B**) centrifuge, (**C**) impurities resulting from the centrifugation, (**D**) rotavapor distillation, and (**E**) column distillation.

The recovery process of the RAP binder was achieved by two processes. The first recovery process involved centrifugation of the solution, followed by rotavapor distillation (RCRD), while the second recovery process involved column distillation of the solution directly without centrifugation (RNCCD). Figure 2 shows the extraction and distillation equipment.

The WEO was blended with the binder before being mixed with the mixture. The blending conditions suggested by Dedene et al. were followed [41]. The temperature of the blend was 150 °C, and the blending time was 0.5 h. The WEO was stirred with a glass rod at the specified temperature and time until the binder was visually homogeneously merged with the binder.

### 3.1. Rheological, Chemical, and Microstructural Testing

The WEO was mixed with the RAP binder in percentages of 0.5, 2.0, and 3.0% by total bitumen weight for the RCRD binder after the extraction and recovery processes of aged bitumen, while it was mixed in percentages 1, 2, and 3% for the RNCCD RAP binder. For the virgin bitumen, RAP binder, and rejuvenated bitumen, the rheological properties were determined via penetration, softening point, and Brookfield viscometer. The chemical and microstructural analyses were conducted through Fourier transform infrared spectroscopy (FTIR), scanning electron microscopy (SEM), and energy-dispersive X-ray (EDX).

The penetration test was performed on the virgin and rejuvenated bitumen according to AASHTO T49 [45]. The softening point test was determined in accordance with ASTM.D36 on both the virgin and rejuvenated bitumen [46]. The Brookfield viscosity of virgin, recovered, and rejuvenated bitumen was measured at three temperatures (135 °C, 150 °C, and 165 °C) according to ASTM D4402M [47].

### 3.2. HMA Testing

The Marshall mix design method—the typical design method used in Egypt for HMA—was used to design four asphalt mixes. These mixes are designated as recycled mix without rejuvenator (R-mix0), recycled mix with 0.5% WEO (R-mix0.5), and recycled mix with 3% WEO (R-mix3). Four tests were conducted in order to assess the rejuvenated RAP mixes’ performance. These tests were Marshall stability, indirect tensile strength test (ITS), dynamic modulus, and flow number [44,48,49,50].

### 3.3. Indirect Tensile Strength Test (ITS)

The tensile strength is a crucial metric for determining the cracking behavior of asphalt pavement. The ITS test is used to indicate the resistance of mixtures to cracking and also assess the moisture susceptibility of asphalt mixes [48]. It is known as one of the most important tests for specifying resistance to various types of failure, such as fatigue cracking [51,52]. The output of the test is the ratio of tensile strength (TSR), which represents the ratio between the sample’s strength before and after water conditioning. The TSR would thus be a significant parameter for rejuvenated mixtures via waste oil. In order to control the test results, it was carried out on samples with almost the same air voids. In this study, the sample sets were chosen to have air voids ranging from 6 to 8%. To achieve the desired range of air voids, a number of trials were performed in order to establish the required number of blows through the compaction process to achieve the target air voids. Two sets of samples were prepared, with three samples for each set. For the first set, no conditioning was applied, while the samples of the second set were first soaked in a water bath to achieve 55–80% saturation. Then, they were conditioned by placing them in a water bath for 24 h at 60 °C. Afterwards, the two sets were immersed in a water bath at 25 °C for at least 1 h before conducting the test.

### 3.4. HMA Dynamic Modulus Testing

**Figure 3:**
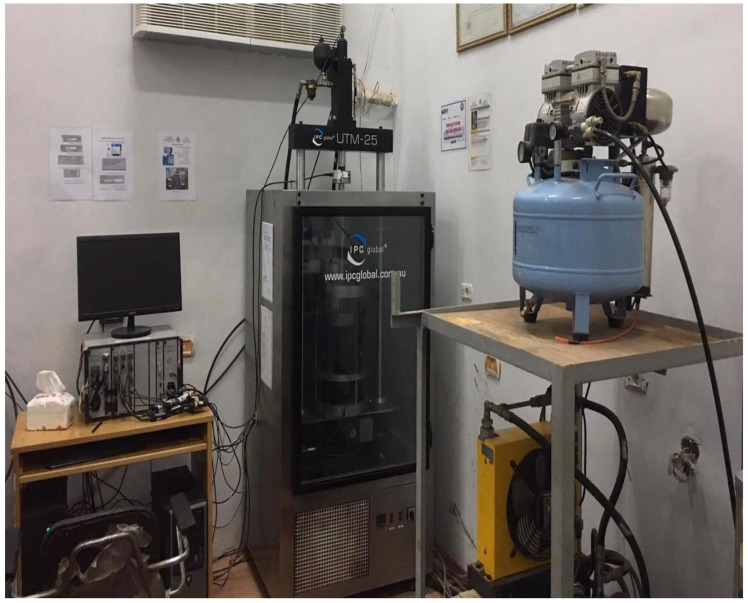
Universal testing machine (UT-25) for dynamic modulus and flow number testing.

**Figure 4:**
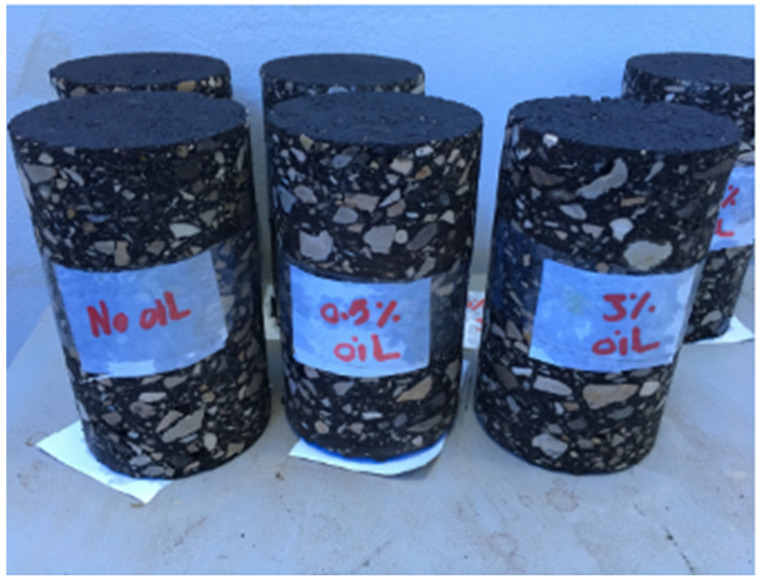
Samples for the E* test after cutting and coring.

The dynamic modulus (E*) of the asphalt mix is the response produced under a continuous sinusoidal loading condition. The complex dynamic modulus is the stress/strain ratio of linearly viscoelastic samples with continuous sinusoidal load in the frequency range [53]. The stiffness of the investigated mixes was evaluated through dynamic modulus testing. A UTM-25 universal testing machine, IPC Global, Boronia, Australia, was used for the test. Figure 3 and Figure 4 display the universal testing machine (UTM-25) used for dynamic modulus/flow number testing and the samples used for the E* test after cutting and coring, respectively.

Air voids in the range of 6–8% were created in compressed cylindrical samples prepared for E* testing in accordance with AASHTO T 342-11, with a 100 mm diameter sample and a 150 mm final height, using a gyratory compactor. This range of air voids was selected because it resembles the initial in situ air voids of pavements just after construction. Typically, each E* or flow number test sample needed around 7.5 kg of mix weight. Two duplicate samples were made for each mix. The E* test was conducted at four temperatures (4.4, 21.1, 37.8, and 54.4 °C) and six different loading frequencies (25, 10, 5, 1, 0.5, and 0.1 Hz).

### 3.5. Flow Number Test

Since the E* test is a nondestructive test, the flow number test was performed on the same sample after the completion of the E* test. The flow number test was performed according to AASHTO TP-79. The specimen was kept in the UTM’s environmental chamber, which was set to 54.4 °C, and the sample was allowed to equilibrate in the environmental chamber for at least 1 h at the test temperature. Under 600 KPa deviator stress and 30 KPa seating stress conditions, the specimen was oriented with the actuator and then exposed to a 0.1 s axial compression load followed by a 0.9 s dwell time every 1.0 s until failure.

## 4. Results and Discussion

### 4.1. Bitumen Rheological Test Results

**Figure 5:**
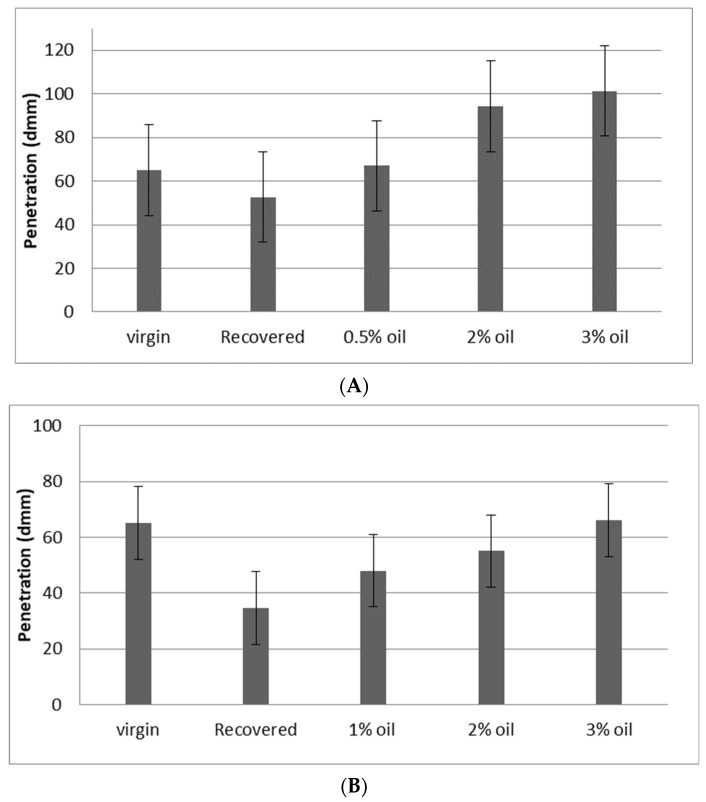
Penetration of virgin, recovered, and rejuvenated bitumen with WEO contents. (**A**) RCRD recovered aged binder; (**B**) RNCCD recovered aged binder.

With regard to penetration, the effect of adding WEO to the recovered RAP binder from the RCRD process and RNCCD process is indicated in Figure 5A,B, respectively. The results show that the RCRD process yielded penetration values higher than those from the RNCCD process. This means that softer binders were obtained using the RCRD process due to the removal of the fines from the recovered bitumen. In both figures, it is noted that the penetration value increased as the WEO increased. For the RCRD and RNCCD binders, 0.5% and 3% WEO were the percentages that achieved the targeted penetration, respectively. The targeted penetration was 60/70, as in the case of virgin bitumen.

**Figure 6:**
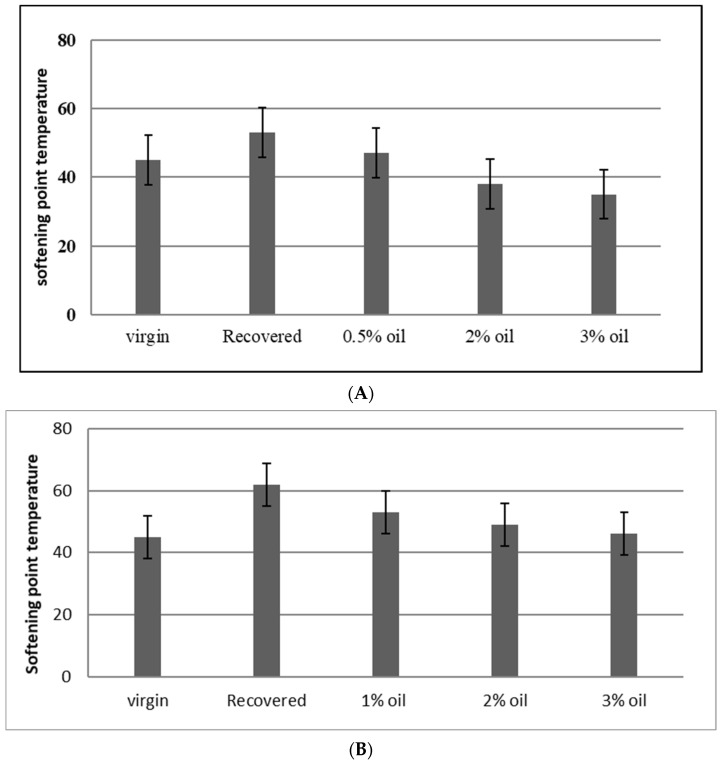
Softening point temperatures for virgin, recovered, and rejuvenated bitumen with WEO contents. (**A**) RCRD recovered aged binder; (**B**) RNCCD recovered aged binder.

Figure 6 shows the softening point temperatures for the virgin, recovered, and rejuvenated bitumen with different percentages of WEO. As shown, the softening point temperature of rejuvenated bitumen was reduced with the addition of WEO. For the RCRD and RNCCD recovered aged binders, 0.5% and 3% WEO were the percentages that achieved the targeted value, respectively. The target of the test was to reach a very close softening temperature to that of virgin bitumen, which is about 45 °C.

**Figure 7:**
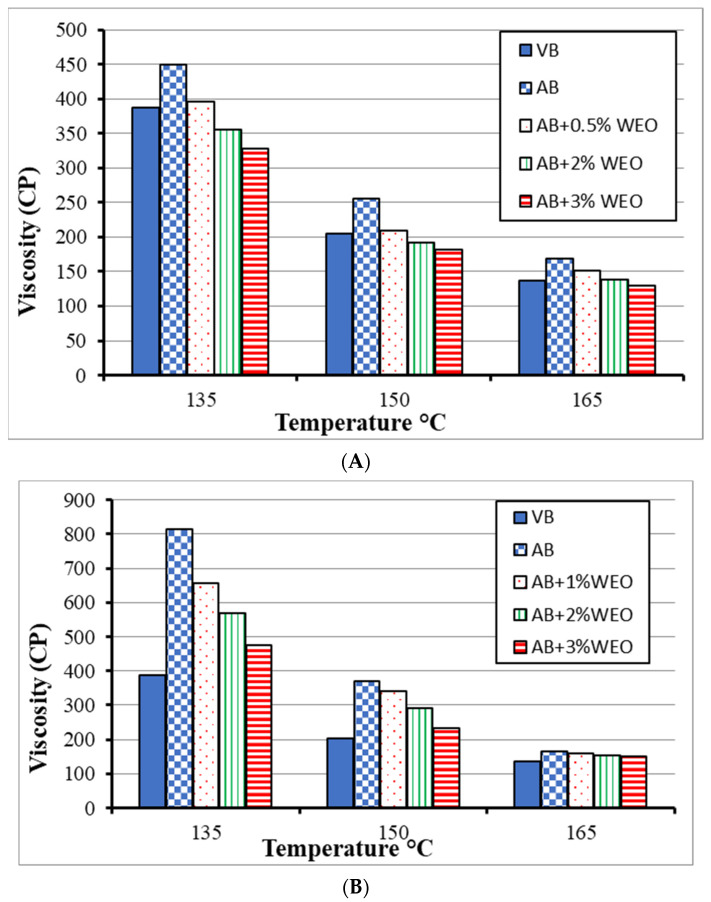
Brookfield viscosity of virgin, recovered, rejuvenated bitumen by WEO contents. (**A**) RCRD recovered aged binder; (**B**) RNCCD recovered aged binder.

Figure 7 indicates the Brookfield viscosity test results for the RCRD recovered binder (Figure 7A) and the RNCCD recovered binder (Figure 7B). The aged bitumen showed the highest viscosity values. When adding 0.5% and 3% WEO to the recovered bitumen for the RCRD and RNCCD recovered binders, respectively, the viscosity values were equivalent to that of virgin bitumen.

Based on the results of the previous binder tests, the optimum contents of WEO that achieved nearly the same properties as those of virgin bitumen were 0.5% and 3% for the RCRD and RNCCD recovered aged binders, respectively. It can be noted that the RCRD process is comparable to the total blending case, in which WEO is added to RAP without any removal of the fines and/or impurities. Based on that, only 0.5% WEO showed an obvious effect on restoring the properties of aged bitumen to that of virgin bitumen. Furthermore, the RNCCD process is comparable to the standard practice case, in which the WEO is added to the extracted bitumen with impurities. The 3% WEO was found to achieve the best results for restoring the virgin characteristics of the aged bitumen. To conclude, it can be stated that the effect of adding WEO to the RCRD aged bitumen appears weaker than that for the processed RNCCD aged bitumen due to the removal of very fine particles.

### 4.2. Microstructural Analysis

**Figure 8:**
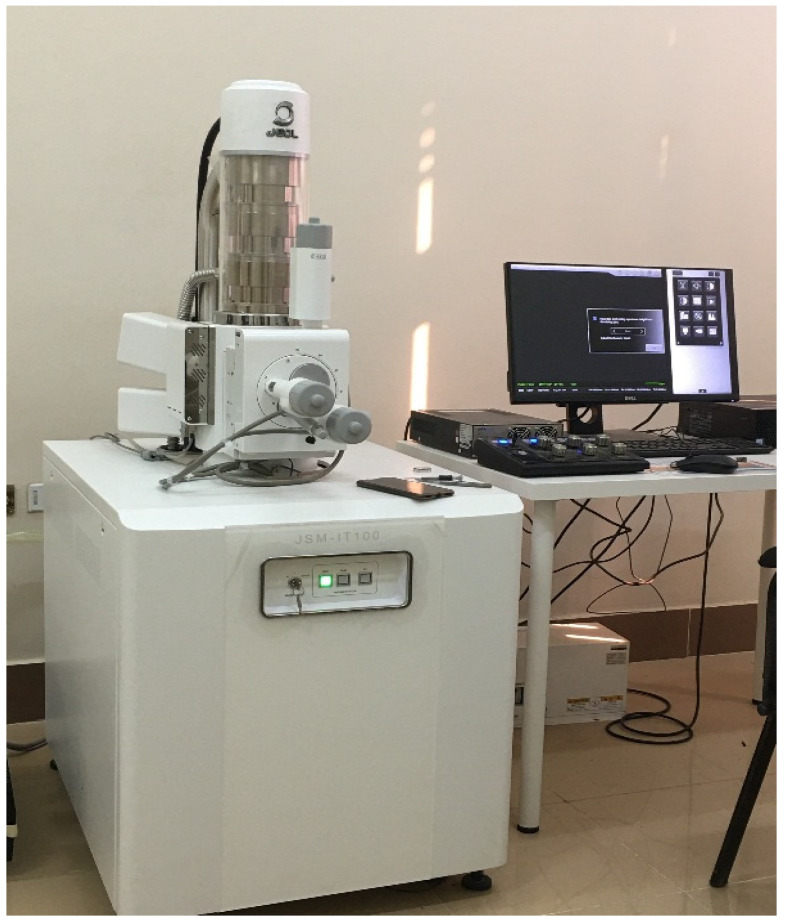
SEM/EDEX Equipment.

The properties of the aged and rejuvenated aged bitumen were examined by scanning electronic microscopy (SEM). Furthermore, quantitative chemical analysis was performed by using a JSM-IT 100, JEOL SEM technologies, Akishima, Japan energy-dispersive X-ray (EDX) spectroscope. Figure 8 shows the SEM/EDX equipment.

**Figure 9:**
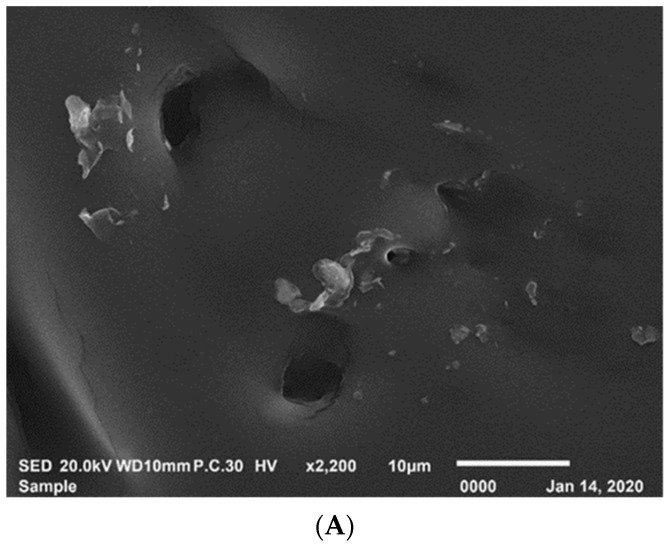

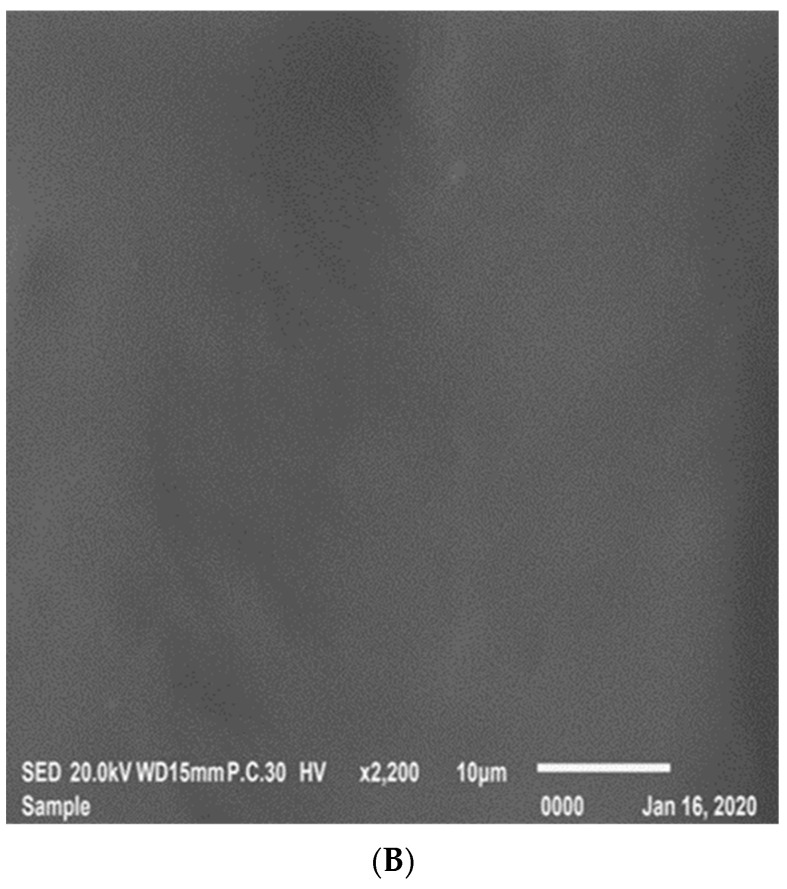
The SEM images of aged bitumen and rejuvenated bitumen. (**A**) Aged bitumen; (**B**) rejuvenated bitumen by the RNCCD process.

SEM is an advanced device that utilizes a focused stream of electrons to produce high-magnification micrographs of the surface sample topography. An electron beam is focused by passing it through a series of magnetic lenses and apertures after it is created by an electron gun. When the beamed electrons impact the sample, they interact with the atoms in the sample, producing a variety of signals. The EDX instrument is attached to the SEM equipment for elemental analysis to show the peaks related to the composition of the tested sample. Figure 9 shows the SEM images of aged and rejuvenated aged bitumen with WEO by the RNCCD process, while Table 4 presents the EDX results.

On the morphological surface of aged bitumen, Figure 9A reveals some unformed fragments with scattered bores that make the surface rough and uneven. The bores and unformed fragments were totally obliterated by the WEO rejuvenator. The main mineral components of the aged bitumen were C (90.49%) and O (7.71%), with a small amount of S (1.32%). The addition of WEO to the recovered binder resulted in an increase in the percent of carbon (C) and aluminum (Al). The increase in the percentage of carbon can be explained by the fact that the main component of motor oil is carbon, which ranges from 73–80% [54]. The presence of aluminum motor body remnants could result in an increase in the percentage of Al [32].

### 4.3. Fourier Transform Infrared Spectroscopy

The FTIR test is an optimization technique for categorizing and quantifying the different types of organic chemical compounds found in different organic substances. The FTIR test was utilized for the binder characterization, as traditional bitumen tests only measure the engineering characteristics of the asphalt binder, whereas the FTIR test reveals differences in the chemical composition between different binders.

Samples of the control and aged binder, in addition to the aged binder modified by WEO, were analyzed using a JASCO FT/IR 6800 apparatus (Madrid, Spain).

**Figure 10:**
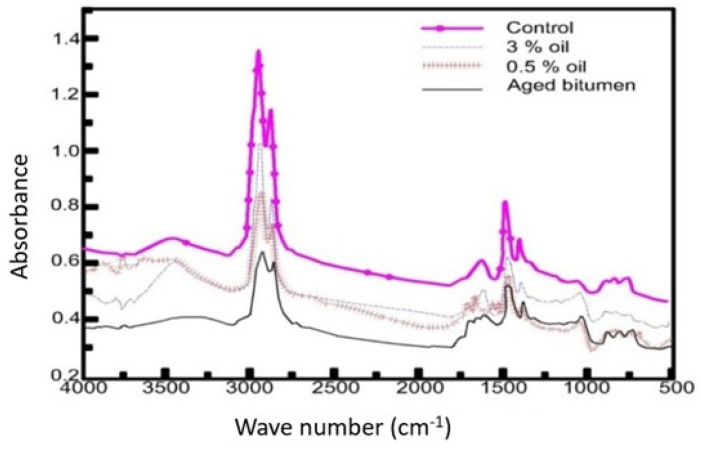
FTIR for virgin bitumen, aged bitumen, and rejuvenated bitumen.

Figure 10 illustrates the FTIR analysis curves of virgin bitumen, aged bitumen, and bitumen rejuvenated by WEO at wavenumbers within 500 and 4000 cm^−1^. It is clear from Figure 10 that the curves of rejuvenated binders are closer to the virgin binder curve than the aged binder curve. This is evidence of the chemical change that occurred in the aged binder due to the addition of the WEO. The appearance of an −OH stretching absorption band is observed in the FTIR spectrum of rejuvenated bitumen at 3396 cm^−1^ and 3448 cm^−1^, respectively. At 2923 cm^−1^ and 2851 cm^−1^, the tensile vibration absorption peak for alkane −CH was improved for the aged bitumen. The rejuvenated asphalt exhibited a significant improvement in C=O and S=O peak areas when compared to virgin bitumen. Table 5 presents FTIR compounds and functional groups for the investigated binders.

Lamontagne proposed a numerical method to quantify the structural indices of carbonyl (IC=O) and sulfoxide (IS=O) compounds, as indicated by Equations (1) and (2) [55,56,57]. It can be said that as the structural indices of sulfoxide or carbonyl compounds decrease, the efficiency of the rejuvenating process improves.

(1) $$I_{C=O}=\frac{\mathrm{Area}\;\mathrm{of}\;\mathrm{carbonyl}\;\mathrm{band}\;\mathrm{centered}\;\mathrm{around}\;1700{\;\mathrm{cm}}^{-1}}{\sum \mathrm{Area}\;\mathrm{of}\;\mathrm{the}\;\mathrm{spectral}\;\mathrm{bands}\;\mathrm{between}\;2000{\;\mathrm{cm}}^{-1}\mathrm{and}\;600{\;\mathrm{cm}}^{-1}}$$

(2) $$I_{S=O}=\frac{\mathrm{Area}\;\mathrm{of}\;\mathrm{sulfoxide}\;\mathrm{band}\;\mathrm{centered}\;\mathrm{around}\;1030{\;\mathrm{cm}}^{-1}}{\sum \mathrm{Area}\;\mathrm{of}\;\mathrm{the}\;\mathrm{spectral}\;\mathrm{bands}\;\mathrm{between}\;2000{\;\mathrm{cm}}^{-1}\;\mathrm{and}\;600{\;\mathrm{cm}}^{-1}}$$

**Figure 11:**
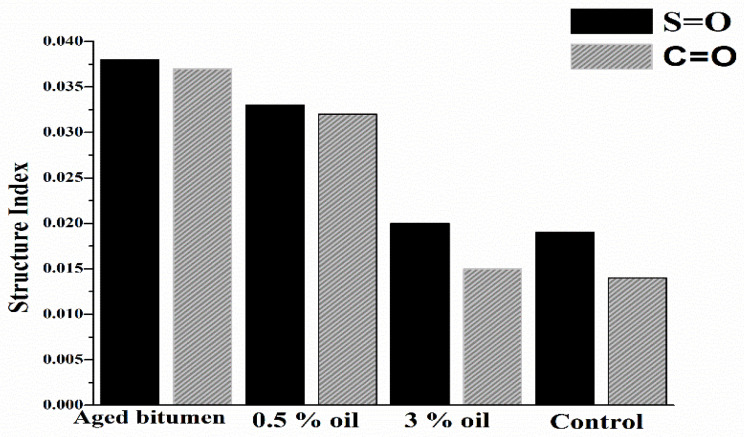
FTIR Structural Indices.

Figure 11 shows the structural indices of the S=O and C=O compounds for all of the investigated binders. Sulfoxides (S=O) and carbonyls (C=O) are widely used to assess binder aging. An increase in S=O and/or C=O compounds is associated with an increase in polar molecules with larger molecular sizes (asphaltenes), leading to more aging [57,58,59]. In Figure 11, it is revealed that adding WEO corresponds to a decrease in all structural indices, which means a decrease in aging. The addition of 3% WEO reduced the structural indices to be identical to the virgin binder. This reduction in sulfoxides and carbonyls is an indicator of a chemical variation in the asphalt binder. When the carbonyl or sulfoxide index decreases, there is a reduction in the quantity of large polar molecules inside the binder (asphaltenes). In other words, the ratio of asphaltenes to maltenes decreased via the transformation from asphaltenes to maltenes inside the asphalt binder. Therefore, this analysis supports using WEO as a rejuvenator for aged binders. These results support the latest research on the suitability of WEO as an effective rejuvenator for aged asphalt binders [26,60].

### 4.4. Marshall Mixture Design for Control and Recycled Mixtures

**Figure 12:**
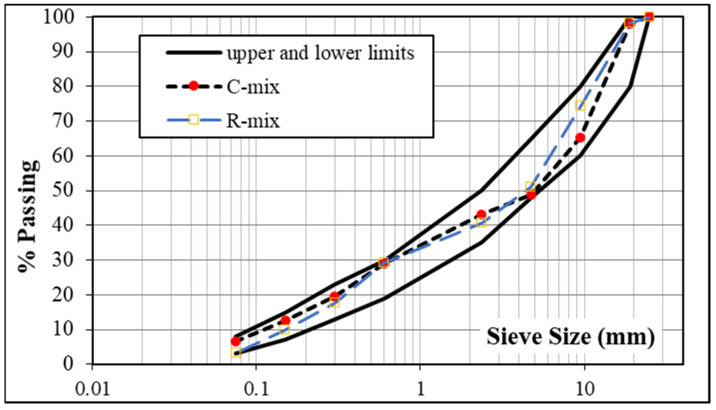
Designed gradation of recycled mix.

All mixes were designed according to the classical Marshall mix design method. For the control and recycled mixes, the aggregates were blended together to achieve the Egyptian specifications for the 4-C wearing surface course [43]. Figure 12 depicts the final gradation, along with the specification limits of the 4-C wearing course for both mixes. The proportions of aggregates 1 and 2 in the control mix were 19 and 32 percent, respectively, whereas the proportions of fine aggregate and mineral filler were 43.5 and 5.5 percent, respectively, by total weight of the mix. As for the recycled mix (R-mix0), the RAP content was 50%, whereas aggregate 1, aggregate 2, fine aggregate, and mineral filler had percentages of 10%, 10%, 25%, and 5%, respectively. It is worth mentioning that the gradations are within the specification limits, and the two gradations were preserved as much as possible, as indicated in Figure 12.

From the Marshall mix design, the Optimum Bitumen Contents (OBC) for the control and recycled mixes were 5.25% and 5.6% (3.1% virgin binder + 2.5% RAP binder), respectively. Triplicate Marshall specimens were produced at the OBC for the C-mix. Moreover, nine Marshall specimens were produced at the OBC for the three different R-mixes (three for R-mix0, three for R-mix0.5, and three for R-mix3). The Marshall variables and volumetric values of all analyzed mixtures are summarized in Table 6. Overall, the Marshall stability met Egyptian requirements for all blends, which stipulate a minimum stability value of 900 kg for large heavily loaded roads [43]. According to the results, the addition of WEO to the recycled mix enhanced its stability and flow compared to the C-mix. In addition, the rejuvenated mixes containing binder recovered from the RCRD recovery process (R-mix3) were better than the rejuvenated mixes containing binder recovered from the RNCCD recovery process (R-mix0.5).

### 4.5. Indirect Tensile Strength Test Results and Discussion

**Figure 13:**
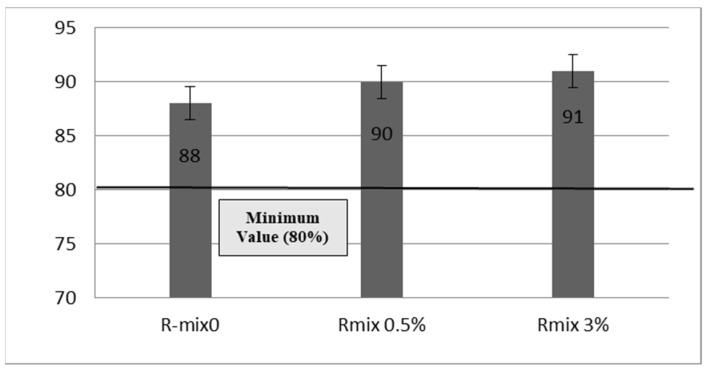
TSR for different asphalt mixes.

The ability of asphalt pavement to withstand moisture is referred to as moisture susceptibility. Moisture penetrated the asphalt mixtures. An indirect tensile strength (ITS) test was carried out to determine the moisture damage resistance. Figure 13 shows the tensile strength ratio (TSR) values for various mixtures, which illustrate the water sensitivity. As the TSR increases, the resistance to moisture damage is improved. It is noted that the rejuvenated RAP mixtures show a substantial improvement compared to the recycled mixture. The TSR values for all mixtures achieved the specifications of the Egyptian Code of Practice, which is 80% min [43].

### 4.6. Dynamic Modulus Results and Discussion

**Figure 14:**
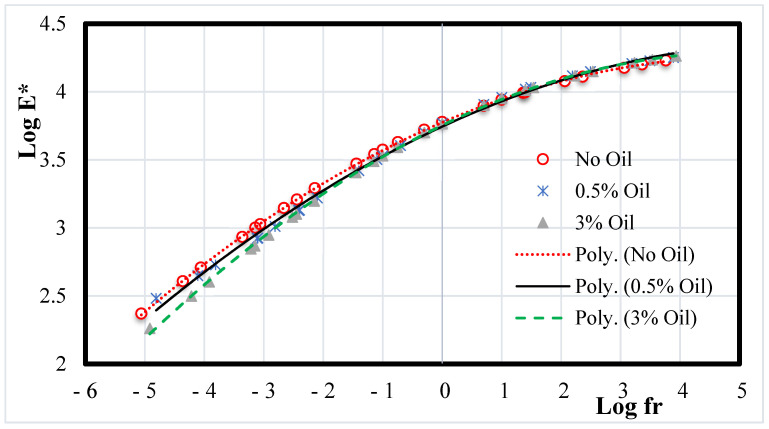
E* Master Curves for Asphalt Mixtures.

Temperature and frequency are the primary determinants of the asphalt mixture’s complex modulus. Figure 14 displays the measured E* values at various testing temperatures and loading frequencies in the form of master curves. The E* master curve is based on the concept of the superposition of time and temperature. Equations (3) and (4) were used to elevate the master curves of the asphalt mixture, in accordance with [61].

(3) $$\log\;\left(E^{*}\right)=\delta \;+\frac{\alpha }{1+{\;e}^{(\beta +{\gamma \mathrm{logf}}_{\begin{array}{c} r)\;\;\; \end{array}}{}^{}}}$$

(4) $$\log\;\left(f_{r}\right)=\log\;\left(f\right)+a_{1\;}\left(T_{r}-T\right)+a_{2\;}{\left(T_{r}-T\right)}^{2}$$

where

E* = asphalt mix dynamic modulus, psi;

f = frequency at test temperature;

T_r_ = reference temperature (21.1 °C);

f_r_ = reduced frequency (Hz) at reference temperature;

T = test temperature, °C;

α, γ, δ, a_1_, a_2_, β = fitting parameters.

As expected, the data show that the values of E* of the recycled mix are higher than those of the rejuvenated mixes. These results support the latest research that reported that adding WEO to RAP softened the aged binder and decreased the dynamic modulus of asphalt mixtures [40,62,63].

### 4.7. Flow Number Test Results and Discussion

**Figure 15:**
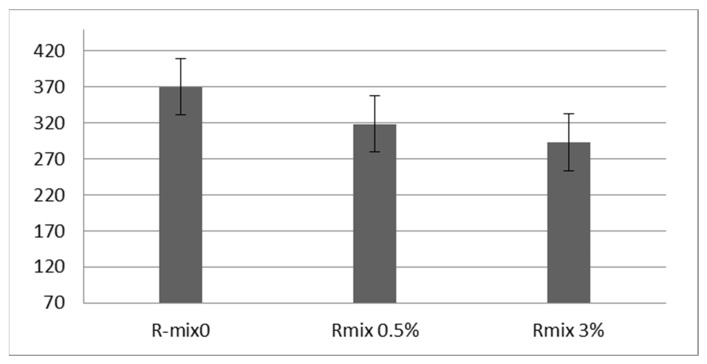
Flow Number Results.

The rutting potential at high temperatures is determined by the flow number. It is known that mixes containing RAP have a better potential for rutting resistance. The flow number values of the investigated mixtures are compared in Figure 15. The figure displays that the flow number decreased for the rejuvenated mixes, meaning that the addition of WEO to the recycled mixes reduced their rutting resistance. These results are consistent with previous studies [62]. This finding indicates that RAP mixes with a rejuvenator are more prone to rutting than RAP mixes without one. However, as the RAP amount increases without a rejuvenator, these mixes will be prone to premature cracking.

## 5. Conclusions

The main goal of the study was to evaluate the performance of typical HMA with a high RAP content using waste engine oil as a rejuvenator. The following conclusions are drawn from the analysis of tests results:

- From the results of the binder tests, the optimum contents of WEO that achieved nearly the same properties as virgin bitumen were 0.5% and 3% for the RCRD and RNCCD processed aged binders, respectively.
- From FTIR results, the addition of WEO reduced the carbonyl (IC=O) and sulfoxide (IS=O) indices to be identical to the virgin binder. The decrease in [IC=O, IS=O] is indicative of a decrease in the amount of asphaltenes (large polar molecules) within the binder.
- The ITS test results revealed that the rejuvenated recycled mixes were more moisture-resistant than the recycled mixes. However, all investigated recycled and rejuvenated mixes achieved the TSR values according to the Egyptian specification requirement, which is 80% as a minimum.
- The addition of WEO to the recycled mix softened the RAP binder, resulting in a reduction in the dynamic modulus of the rejuvenated mixes, making them more resistant to fatigue cracking. However, the flow number decreased for the rejuvenated mixes, meaning reduced rutting resistance.

## Figures and Tables

**Table 1:** Comprehensive review of literature studies on the effects of utilizing waste engine oil on the performance of asphalt binders and/or mixtures.

Reference	Type of Waste Oil (Source)	% of Waste Oil to Binder Content	Binder Grade	Experimental Testing	Effect on the Mix/Binder
[35]	WEO (NA)	7	NA	Indirect tensile strength (ITS) Durability Resilient modulus (MR)	Using 7% WEO improved the performance of recycled mix by up to 40% Moisture damage increases with increases in RAP% and rejuvenators
[32]	WEO (local auto service shops)	2, 3, 3.5, and 4	60/70	Penetration (Pen), Brookfield viscosity (BV), softening point (SP) DSR, BBR, FTIR, SEM- EDX Marshall stability ITS	Low-temperature thermal cracking improved Improved stability Improved moisture resistance
[36]	WEO (local auto repair shop)	1, 2, 3, 4, and 5	60/70	Pen, SP, viscosity (V), ductility FTIR, SEM DSR	Improved the physical properties of aged asphalt to the normal level Improved workability
[9]	WEO (vehicles workshop)	1, 2, and 3	40/50	Ductility, Pen, SP, flash point (FP), specific gravity (SG), kinematic viscosity (KV), loss on heating FTIR	3% WEO restored the properties of the aged asphalt to its original Enhanced viscosity Enhanced temperature susceptibility Enhanced aging resistance
[37]	WEO (garage)	4 and 8 *(bitumen modifier)	60/80	FTIR Gel permeation chromatography Rotational viscosity (RV) DSR Gas chromatography–mass spectrometry	WEO had a detrimental impact on asphalt rutting resistance while having a beneficial impact on fatigue behavior The phase angle increased as the complex modulus decreased WEO reduced binder viscosity and construction temperatures
[38]	WEO (industrial organizations)	5.4	50/70	V, FP, Pen, SP, SG Marshall stability ITS Rolling thin film oven (RTFO)	Improved the workability Aging indices of rejuvenated mixtures improved
[39]	WEO (workshop)	6	80/100	Pen, SP, V, DSR	The rutting resistance increased while the softening point decreased The addition of WEO to the modified asphalt binder reduced the stiffness The viscosity decreased with the increase in WEO
[40]	WEO (automobile engine oil)	2 and 5	PG 64–22	ITS Beam fatigue test Asphalt pavement analyzer test MR	Increased stiffness Decreased OAC Improved the workability Improved the performance of HMA Improved fatigue resistance of HMA
[14]	WEO (Michigan Tech motor pool)	4 and 8	PG 58–28	ITS FTIR	Increased rutting Reduced indirect tensile strength FTIR test revealed a decrease in the structural indices of sulfoxides and carbonyl, confirming an increase in the proportion of maltenes
[41]	WEO (Michigan Tech motor pool)	4, 8	PG 70–22	DSR and RV	DSR and rotational viscometer test results showed that the stiffness improved
[42]	WEO (workshops)	10	80/100	Ductility, SG, Pen, SP, FP, V, loss on heating	The addition of 10% WEO to aged bitumen can restore its properties, especially viscosity and standard penetration grade

* Bitumen modifier; NA = not available; Pen = penetration; BV = Brookfield viscosity; SP = softening point; RV = rotational viscosity; V = viscosity; SG = specific gravity; FP = flash point.

**Table 2:** Virgin and aged binder properties.

Test	Results	Specification Limits
	60/70 asphalt	
Softening point, °C	45	[45–55]
Penetration	65	[60–70]
Specific gravity	1.02	-
Viscosity (135 °C), centipoise	387	[+320]

**Table 3:** Gradation of coarse aggregate, sand, mineral filler, and RAP.

Sieve Size (Inch)	Coarse Aggregate (Passing %)		Sand (Passing%)	Mineral Filler (Passing %)	RAP
	Coarse Aggregate (1)	Coarse Aggregate (2)			
1	100	100	-	-	100
3/4	100	94.0	-	-	88.6
3/8	80.1	3.3	-	-	46.4
No. 4	3.5	-	98.0	-	24.1
No. 8	-	-	86.6	-	14.0
No. 30	-	-	54.1	100	5.3
No. 50	-	-	32.2	99.2	2.4
No. 100	-	-	16.8	98.2	
No. 200	-	-		93.6	

**Table 4:** The EDX results of aged bitumen and rejuvenated bitumen.

Sample	Source	Formula	Mass%	Atomic%
Aged Bitumen	CaCO_3_	C	85.27	90.48
	SiO_2_	O	9.67	7.71
	Al_2_O_3_	Al	0.38	0.18
	FeS_2_	S	3.32	1.32
	Fe	Fe	1.37	0.31
Bitumen rejuvenated by WEO	CaCO_3_	C	96.02	98.42
	FeS_2_	S	3.25	1.25
	Al_2_O_3_	Al	0.74	0.34

**Table 5:** Fourier transform infrared spectroscopy functional groups.

Virgin Bitumen		RAP Binder		RAP Binder + 0.5% Oil		RAP Binder + 3% Oil	
Wave Number	Function Group	Wave Number	Function Group	Wave Number	Function Group	Wave Number	Function Group
3431	−(OH)	3313	−(OH)	3396	−(OH)	3448	−(OH)
2918	CH-Alkane	2922	CH-Alkane	2922	CH-Alkane	2922	CH-Alkane
2853	CH-Alkane	2851	CH-Alkane	2846	CH-Alkane	2849	CH-Alkane
1711	C=O	1688	C=O	1693	C=O	1707	C=O
1599	C=C	1597	C=C	1592	C=C	1590	C=C
1455	C-H Bending	1455	C-H Bending	1455	C-H Bending	1455	C-H Bending
1024	S=O	1031	S=O	1021	S=O	1027	S=O

**Table 6:** Summary of Marshall parameters and volumetric properties of the investigated mixes.

Mix Type	OBC % (%V Bitumen + %RAP Binder)	Stability kg	Flow mm	Bulk Specific Gravity	Theoretical Specific Gravity	Va %	VMA %	VFA %	Vbe %
C-mix	5.25	1050	3.24	2.362	2.460	3.90	15.40	73.0	11.50
R-mix0	5.6 (3.1% + 2.5%)	1434	2.80	2.348	2.422	3.05	15.23	80.2	12.2
R-mix0.5		1223	2.80	2.348	2.422	3.07	15.28	79.9	12.2
R-mix3		1030	3.20	2.349	2.422	3.01	15.23	80.0	12.2
Limits	4:7.5	Min 900	2:4	--	--	3:5	Min 15	--	--

Va = air voids; VMA = voids in mineral aggregate; VFA = voids filled with bitumen; Vbe = volume of effective bitumen.
